# Metagenome-assembled genome distribution and key functionality highlight importance of aerobic metabolism in Svalbard permafrost

**DOI:** 10.1093/femsec/fiaa057

**Published:** 2020-04-17

**Authors:** Yaxin Xue, Inge Jonassen, Lise Øvreås, Neslihan Taş

**Affiliations:** 1 Computational Biology Unit, Department of Informatics, University of Bergen,Thormøhlensgt 55 N-5008, Bergen, Norway; 2 Department of Biological Sciences, University of Bergen, Thormøhlensgt 53 N-5020, Bergen, Norway; 3 University Center in Svalbard, UNIS, N-9171, Longyearbyen, Norway; 4 Ecology Department, Earth and Environmental Sciences Area, Lawrence Berkeley National Laboratory, 1 Cyclotron Road, Berkeley, CA 94720, USA; 5 Environmental Genomics and Systems Biology, Biosciences Area, Lawrence Berkeley National Laboratory, 1 Cyclotron Road, Berkeley, CA 94720, USA

**Keywords:** Svalbard, permafrost, microbiome, metagenome-assembled genomes, aerobic metabolism

## Abstract

Permafrost underlies a large portion of the land in the Northern Hemisphere. It is proposed to be an extreme habitat and home for cold-adaptive microbial communities. Upon thaw permafrost is predicted to exacerbate increasing global temperature trend, where awakening microbes decompose millennia old carbon stocks. Yet our knowledge on composition, functional potential and variance of permafrost microbiome remains limited. In this study, we conducted a deep comparative metagenomic analysis through a 2 m permafrost core from Svalbard, Norway to determine key permafrost microbiome in this climate sensitive island ecosystem. To do so, we developed comparative metagenomics methods on metagenomic-assembled genomes (MAG). We found that community composition in Svalbard soil horizons shifted markedly with depth: the dominant phylum switched from *Acidobacteria* and *Proteobacteria* in top soils (active layer) to *Actinobacteria*, *Bacteroidetes*, *Chloroflexi* and *Proteobacteria* in permafrost layers. Key metabolic potential propagated through permafrost depths revealed aerobic respiration and soil organic matter decomposition as key metabolic traits. We also found that Svalbard MAGs were enriched in genes involved in regulation of ammonium, sulfur and phosphate. Here, we provide a new perspective on how permafrost microbiome is shaped to acquire resources in competitive and limited resource conditions of deep Svalbard soils.

## Teaser

Two novel computational methods were developed to conduct a deep metagenomic analysis of Svalbard permafrost samples, resulting in previously unreported trends in permafrost, especially the importance of aerobic metabolisms.

## INTRODUCTION

Permafrost covers nearly one quarter of Earth's terrestrial surface and stores an estimated amount of 20%–50% of global soil organic matter (SOM) (Schuur *et al*. 2008; Tarnocai *et al*. 2009). In the Northern Hemisphere as much of 24% of the soil is permanently frozen (Alley *et al*. 2007). These ecosystems are proposed to provide a unique environment for cold-adapted microorganisms and shown to contain highly diverse microbial communities (Jansson and Taş 2014). Global warming is expected to have its largest impact through thawing of permafrost and the scale of this impact depends strongly on the amount and vertical distribution of ground ice (Kokelj *et al*. 2017). During the past decade, with steadily rising temperatures, permafrost thaw has accelerated across the Arctic areas (Hayes *et al*. 2014). The effect of large-scale permafrost thaw becomes a serious concern as it may increase the microbial activity leading to SOM degradation and release of more greenhouse gases (GHGs) – such as carbon dioxide (CO_2_) and methane (CH_4_) – hence contributing to further global warming (Jansson and Taş 2014). Therefore, it is highly relevant to characterize the bacterial community residing in the permafrost in terms of species composition and its metabolic and functional potential. Advances in next-generation sequencing (NGS) has expanded our ability to characterize the microbiome and investigate potential metabolisms from permafrost samples. For example, metagenomics was critical to identify substantial functional and compositional differences between active layer (AL: experiences seasonal thaw-refreeze) and permafrost layer (PL: constantly frozen for more than two consecutive years), which showed that transition from frozen to thaw state stimulates SOM-degrading microbes (Mackelprang *et al*. 2011). While metagenomics continues to transform our understanding of microbial functions upon thaw (Jansson and Taş 2014; Hultman *et al*. 2015; Woodcroft *et al*. 2018) most of our current knowledge is still based on studies that are focused on 16S rRNA gene-sequencing analysis (Wilhelm *et al*. 2011; Gittel *et al*. 2014; Koyama *et al*. 2014; Deng *et al*. 2015; Mackelprang *et al*. 2016a). These studies are informative for describing species or groups of species in a community permafrost microbiome but is less suited for exploring functional potential and novel species distribution (Knight *et al*. 2018).

The Svalbard archipelago is a unique permafrost environment located at Arctic–Atlantic Ocean border. About 60% of the land is covered by glaciers but remainder periglacial environment contains the largest permafrost area in Europe outside of Russia. In contrast to other regions with extensive permafrost areas, such as Siberia and Northern Alaska, permafrost in Svalbard is presumably of young age (i.e. Holocene) specifically at low altitude areas around central Spitsbergen. However, high altitude permafrost in Svalbard may represent an exception to this (Humlum, Instanes and Sollid 2003). The North Atlantic Current dampens polar influence in Svalbard where especially winter temperatures could be up to 20°C higher than similar latitudes in Russia and Canada (Humlum, Instanes and Sollid 2003). As a result, permafrost in Svalbard is proposed to be more sensitive to changes in temperature and soil thickness (Humlum, Instanes and Sollid 2003). Research in Svalbard provides an opportunity to study the immediate effects climate change and permafrost thaw. Svalbard had been a focal point of studying glacial, subglacial (recently deglaciated), cryoconite sediments (Kastovská *et al*. 2005; Edwards *et al*. 2011) and tundra microbiomes (Tveit *et al*. 2013; Schostag *et al*. 2015; Bang-Andreasen *et al*. 2017). The Arctic tundra in Svalbard contains diverse microorganisms which are active throughout the winter despite the freezing conditions (Schostag *et al*. 2015). Peatlands of Svalbard are shown to be inhabited by microbes governing biogeochemical cycles through hydrolysis of plant polymers, fermentation, methanogenesis and methanotrophy where *Actinobacteria* was identified as a key phylum carrying out SOM degradation (Tveit *et al*. 2013). However, in comparison with other soils, our knowledge of the Svalbard permafrost microbiome is limited. In a previous publication from Adventdalen Valley permafrost, we showed that PL were significantly different from the AL, where microbial community structure changed strongly with depth and *Actinobacteria* were identified as the dominant microbial phylum of PL via 16S rRNA gene sequencing (Müller *et al*. 2018). However, others also showed that *Actinobacteria*, *Bacteroidetes*, *Firmicutes* and *Proteobacteria* are major parts of the microbiome (Bang-Andreasen *et al*. 2017) of near surface permafrost at this location suggesting that Adventdalen Valley permafrost is likely to have a highly heterogeneous composition.

In this study, we investigated the microbial composition and functional potential through a permafrost core from Svalbard's Adventdalen Valley in order to determine key microbial functional potential. Although metagenomics provides holistic view to microbial functions from largely unculturable permafrost microbiome (Mackelprang *et al*. 2016b), several aspects of bioinformatic analysis remain challenging. For example, we are still lacking an effective and robust workflow for recovering quality metagenome-assembled genomes (MAGs) from the permafrost communities due to the large complexity and heterogeneity present in these soils. More importantly tools enabling systematic comparison among metagenomes by taking full advantage of data and maximize the information driven from samples, are urgently needed. To address these issues, we developed computational tools to aid high-quality MAG recovery and to identify key functions through comparative functional analysis. We aimed to capture the variances in microbial composition and trends in functional potential throughout the depth profile (AL to PL). We hypothesized that (i) phylogenetically related MAGs resides in PL where (ii) SOM-degradation pathways in key permafrost microbiome are represented by mix of aerobic and anaerobic processes.

## MATERIALS AND METHODS

### Sample collection

Soil samples were obtained from an ice-wedge polygon site in the Adventdalen Valley in Svalbard, Norway (78.186 N, 15.9248E) in 2011. Adventdalen represents a classic high-arctic fjord-valley, which are sediment filled paleo fjords characteristic to formerly glaciated mountain coastal areas. Detailed description and procedures for core collection and characterizing soil samples were described previously (Müller *et al*. 2018). In short, the permafrost core was collected in by automated drilling in April 2011 in Adventdalen, Svalbard. The total length of the core was 198 cm, and the core was immediately frozen at −20°C, until further processing. The entire core was scanned by X-ray computed tomography (CT) imaging, and cut into 1–2 cm slices using saw blades sterilized with ethanol. To remove potential surface contaminants (Bang-Andreasen *et al*. 2017) from the core fragments the outermost 2 cm were cut off using sterile blades. Based on the results from the temperature loggers, CT scanning and water content of the permafrost core (Müller *et al*. 2018) active and PL depths were decided. Five fragments, one from AL and four PL, with different depths AL1 (7 cm), PL1 (110 cm), PL2 (122 cm), PL3 (135 cm), PL4 (170 cm) below the soil surface were subjected to metagenomics analyses. Both AL and PL soils were acidic (pH: 4.6 AL; pH: 4.5–5.0 PL) and contained 1.3%–1.7% C gr soil (Müller *et al*. 2018).

### Metagenomic sequencing, recovery and refinement of MAGs

**Figure 1.**
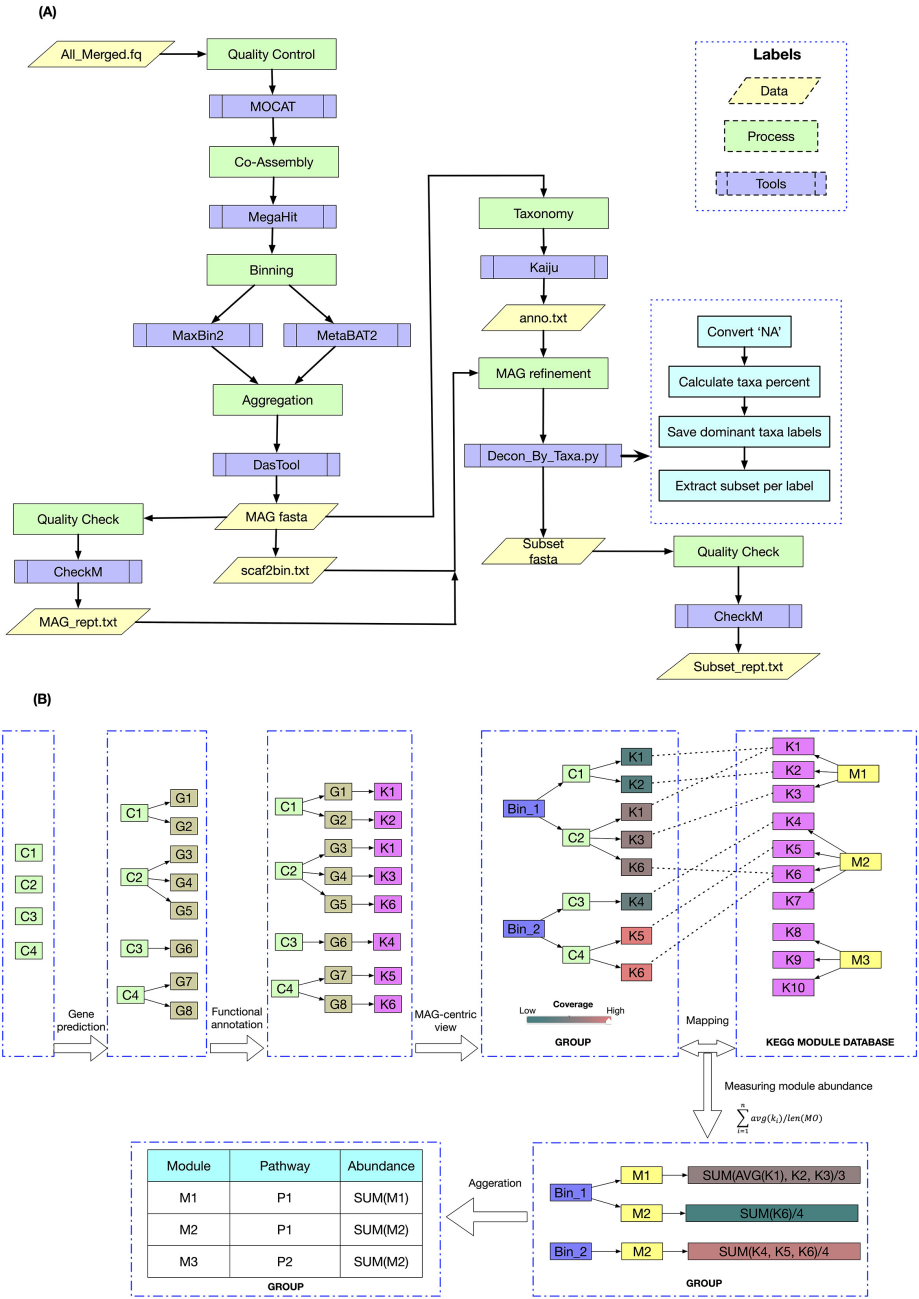
Overview of Svalbard permafrost bioinformatic strategies. **(A)**, An improved workflow for MAG recovery and refinement. The entire workflow includes several steps and tools, including quality control, co-assembly, binning, aggregation, quality check and MAG refinement. See details in methods. **(B)**, A schematic illustration for coverage-based functional analysis in a MAG-centric view. Each contig (C), contains multiple genes (G) that can be annotated with KEGG Orthology (K) and linked with KEGG Module (M) database. Coverage can be used as a quantitative measure for each KEGG Module hence allowing analysis of trends of increasing or decreasing representation between and within sample set (Table 1). SUM: Summation. AVG: Average.

DNA was extracted and libraries prepared using procedures described previously (Xue *et al*. 2019). Metagenome sequencing was performed using the Illumina HiSeq 2500 instrument to acquire 150 bp paired-end sequences, generating around 20Gbp per sample after quality control (trim and discard low-quality sequences) with MOCAT2 v2.0.0 (Kultima *et al*. 2016). The analysis workflow used here organizes several bioinformatic scripts to recover and refine MAGs (Fig. 1A). Firstly, all quality controlled reads were co-assembled with MEGAHIT v1.1.3 (Li *et al*. 2015). Two binning tools, MaxBin2 v2.2.5 (Wu, Simmons and Singer 2016) and MetaBAT2 v2.12.1 (Kang *et al*. 2015), were used and output bins were further dereplicated and aggregated with DASTool v1.1.10 (Sieber *et al*. 2018). The checkM v1.0.11 (Parks *et al*. 2015) was used to determine completeness and contamination of MAGs. We observed that a large portion of bins had a high contamination percentage even after using DASTool. To improve the quality of MAGs, we developed a script, called ‘Decon_MAG_by_taxa.py’, that will subset each bin into collections of contigs from the same taxonomic classification. In theory each bin represents an individual genome with single-taxon annotation. However, in practice bins contain contigs from other taxa due to the complexity of microbial communities. Yet it is possible to remove those contaminations by parsing their taxonomic classification. First, each bin was annotated with Kaiju v1.6.2 (Menzel, Ng and Krogh 2016) using default parameters utilizing the NCBI nr database to classify each contig into a taxonomic rank, from phylum to species. Then script extracts contigs with the same taxonomic classification at each rank and generates multiple subsets of fasta files corresponding to each rank.

By default, Kaiju will return a ‘NA’ if it cannot find a taxonomic classification at certain ranks, which results in many ‘NA's at lower rank and loss of hierarchical taxonomic structure while contamination may happen in any rank. To maximum utilize the taxonomic annotation, here we considered ‘NA’ in Kaiju annotation as a special taxonomic rank, and sustained the hierarchical structure under the following rules: (i) when ‘NA’ observed in a non-phylum level, a label is generated via combining higher taxonomic rank information with ‘_NA_’ denotation as a rank identifier (P: Phylum, C: Class, O: Order, F: Family, G: Genus, S: Species), (ii) if ‘NA’ appeared at the phylum level a label is generated as ‘P_NA’. For example, if a contig is annotated as: ‘C1; Proteobacteria; Alphaproteobacteria; Rhizobiales; NA; NA; Unknown species’, then it will be converted to: ‘C1; Proteobacteria; Alphaproteobacteria; Rhizobiales; Rhizobiales_NA_F; Rhizobiales_NA_F_NA_G; Unknown species’. Later, the script calculates the percentage of every taxa label in each rank and keeps labels whose percentage were higher than a user-defined threshold (default = 0.5). As the script provides multiple subsets of fasta corresponding to different ranks for each bin, the user can run CheckM with all of these subsets and evaluate the best tradeoff between completeness and contamination. More detailed description of our MAG refinement method is available at: https://github.com/yxxue/Recovery-and-refinement-of-MAGs-for-permafrost-metagenome.

MAGs were annotated to a taxonomic rank based on Kaiju and GTDB-Tk v0.3.3 (Parks *et al*. 2018) annotation. For each sample, we aligned sequence data against all refined MAGs using BBMAP v37.36 (https://sourceforge.net/projects/bbmap/) with default parameters. The relative abundance of each MAG was calculated by aggregating the mapping ratio of contigs pertained to this MAG. RAST annotations for the MAGs are publicly available at KBase narrative (Arkin *et al*. 2018): https://narrative.kbase.us/narrative/ws.50152.obj.370 (KBase account required).

### Coverage-based functional analysis in a MAG-centric view

#### Normalization coverage

To perform quantitative comparative analysis, we utilized a normalization strategy – TPM (Transcripts Per Kilobase Million) – which is commonly used in normalizing gene expression in RNA-seq analysis (Wagner, Kin and Lynch 2012). Our normalization method consists of three steps. Firstly, we considered coverage of contigs as RPK value of contigs, as coverage represent the number of mapped reads divided by the length of the contig, which is analogous to be the concept of PRK value. Second, we calculated the ‘per million’ scale factor by dividing total mapped read counts with 1 million in each sample. For example, the mapped reads count in AL1 was 9 171 534, thus the scaling factor in AL1 would be 9.171534 (9171,534/1000,000). Finally, coverage was normalized by dividing corresponding scaling factor, respectively.

#### Definition of groups

We pre-defined several groups combining the coverage patterns with geographical significance (Table 1). To capture the distinct variation in terms of coverage profiles among contigs, we chose median of the normalized coverage as a global threshold to classify contigs and removed low coverage contigs (LO). Active layer (AL) was simple case in our data sets since there was only one sample representing the active layer while we found that coverage distribution in PL were more complicated and needed to be considered separately: some contigs were only present in specific samples, while others appeared in full or in part in all PL samples. Therefore, we defined three groups for PL samples: PL_Pi(only present in specific samples), PL_SUB (present in some of the samples) and PL_ALL(present in all samples). Besides, we derived contigs that had a strong correlation (0.9) between depth and coverage from PL samples, namely KI and KD. Group BO represented the ubiquitous contigs in Svalbard AL and PL, remaining contigs were assigned to UN (unknown).

**Table 1.** Definition of sample groups. AL: normalized coverage in active layer, PL: normalized coverage in permafrost layer samples. TH: threshold (median of normalized coverage). DEPTH (cm under surface): 110, 122, 135, 170. CORR: Pearson correlation.

Groups	Definition	Criteria
AL	Presence in AL	AL > = TH and ALL(PL) < = TH
BO	Presence Both in AL and PL	AL > = TH and ALL(PL) > = TH
LO	Absence Both in AL and PL	AL < = TH and ALL(PL) < = TH
PL_SUB	Presence in subset (2 or 3) PL	AL < = TH and SUB(PL) > = TH
PL_ALL	Presence in all PL	AL < = TH and ALL(PL) > = TH
PL_Pi	Presence in unique PL (P1, …,P4)	AL< = TH and UNIQUE(PL_Pi) > = TH
KI	Increasing trend in PL_ALL or PL_SUB	In (PL_ALL or PL_SUB) and CORR(PL, DEPTH) > = 0.9
KD	Decreasing trend in PL_ALL or PL_SUB	In (PL_ALL or PL_SUB) and CORR(PL, DEPTH) < = −0.9
UN	Unknown groups	Others

#### Calculating KEGG Module abundance of MAGs

We considered each MAG as an independent unit and normalized coverage was used to represent KEGG Orthology (KO) abundance. An illustration of our strategy is shown in Fig. 1B. First, we used Prodigal v2.6.3 (Hyatt *et al*. 2012) with meta procedure to predict genes for all MAGs. Predicted protein file was then uploaded to perform KO annotation using GhostKOALA (Kanehisa, Sato and Morishima 2016). Later, we converted the gene-based KO annotation to a MAG-centric hierarchical structure and calculated KEGG module abundance. KEGG Module (MO) is a collection of KOs, which represents tight functional components with a clearer biological significance comparing with KO identifiers. In each MAG, abundance of KEGG Modules (MOs) was calculated by summing the average existing KO and then dividing by total number of KO identifiers in this module. MO abundance in each group was measured by aggregating MO abundance of all MAGs presented at each group, respectively. As the demonstration shown in Fig. 1B, M1 consists of 3 KO (*K1-K3*) and M2 of 4 KO (*K4-K6*). Bin_1 includes two weighted (normalized coverage) contigs with 5 KO: C1 (*w_1_K1*, *w_1_K2*) and C2 (*w_2_K1*, *w_2_K3, w_2_K6*). Based on the definition of MAG, we suppose that contigs in the same MAG are able to share their KO: we further use average if there are multiple hits for identical KO in the same MAG. Therefore, M1 abundance in Bin_1 is: *SUM(AVG(w_1_K1, w_2_K1), w_1_K2, w_2_K3) / 3*. Similarly, only *K4* in M2 is detected in Bin_1 while M2 consists of 4 KO, thus M2 abundance in Bin_1 is: *SUM(w_2_K6) / 4*. Finally, M1 abundance in this group is simply aggregating all M1 abundance of each MAG. A detailed demonstration of performing our coverage-based analysis and source code are available at https://github.com/yxxue/Coverage-based-functional-analyisis-in-a-MAG-centric-view.

## RESULTS

### Unique MAGs become abundant with depth in Svalbard permafrost

**Figure 2.**
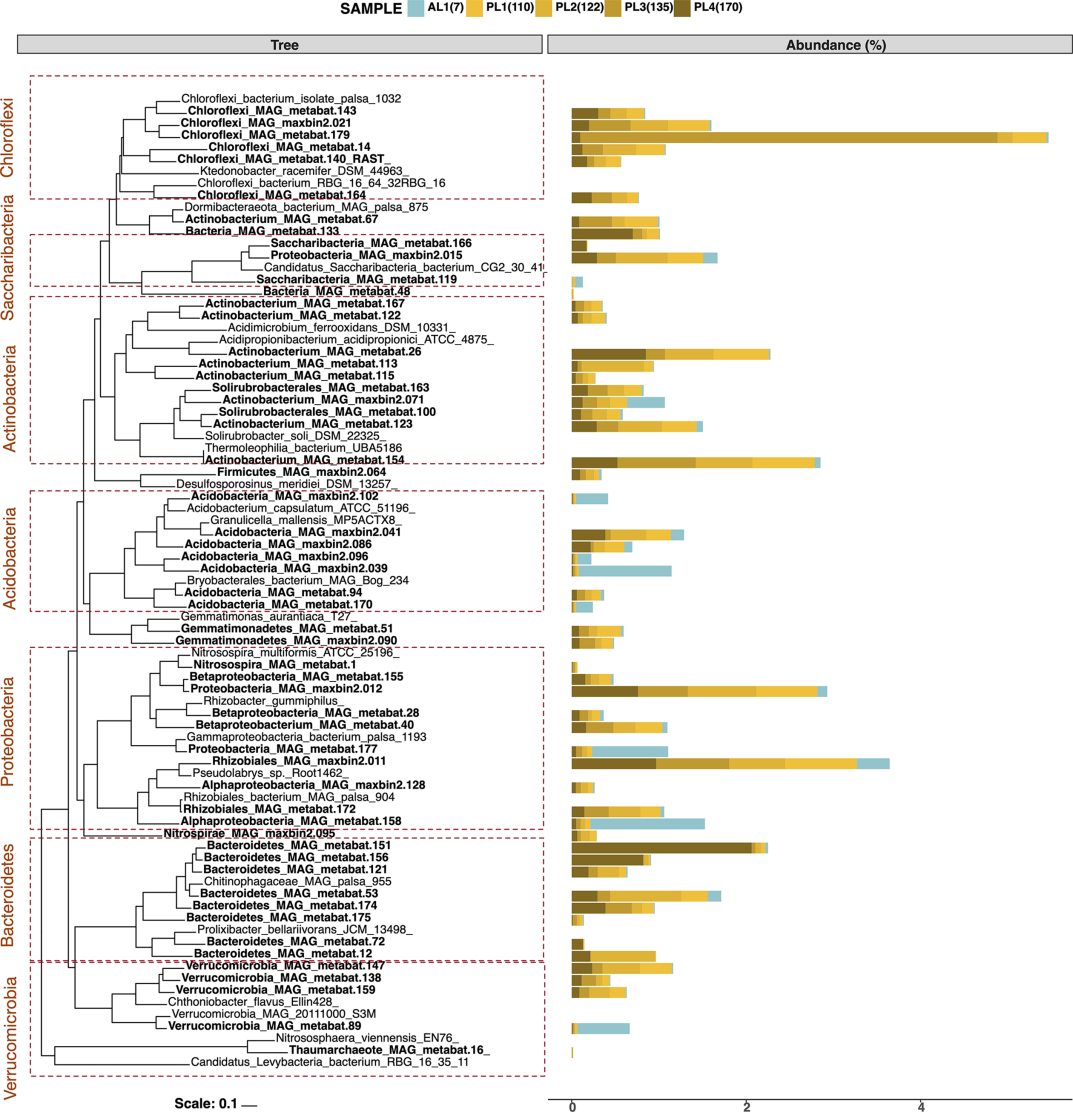
The relative abundance of MAGs shifts between samples. Percent MAG abundance in five soil layers, one active layer (AL, blue color) and four permafrost layers (PL, yellow to brown color), with different depths below the soil surface are shown: AL1 (7 cm), PL1 (110 cm), PL2 (122 cm), PL3 (135 cm) and PL4 (170 cm). Maximum likelihood phylogenetic tree was constructed by using 49 highly conserved COG families from publicly available genomes.

We reconstructed 56 MAGs from 13 phyla, including 8 high, 44 medium and 4 low-quality draft in accordance with MIMIG standards (Bowers *et al*. 2017). In total, the analyzed MAGs constituted on average 11.3% of the reads obtained for each sample (min. 7.1% and max. 13.4%). In this location, we found several MAGs belonging to *Actinobacteria, Proteobacteria, Bacteroidetes, Acidobacteria* and *Chloroflexi* to be most abundant (Fig. 2). Additionally, MAGs belonging to *Verrucomicrobia, Saccharibacteria, Gemmatimonadetes, Firmicutes, Nitrospirae, Thaumarchaeota, candidate phylum Dormibacteraeota (AD3)* and *candidate phylum Levybacteria* were found in lower abundance. We did not recover any methanogenic archaea in this location. Detailed description of MAGs were published previously (Xue *et al*. 2019). MAGs showed low similarity to publicly available genomes (Table S1, Supporting Information) suggesting that they represent novel species. We also compared these MAGs to microbiomes of recent stable isotope probing showing activity at subzero conditions (Tuorto *et al*. 2014; Gadkari *et al*. 2019). Svalbard MAGs were distantly related to these novel populations and showed 75%–88% similarity on 16S rRNA genes (Table S2).

Microbial community composition based on changes in MAG abundance showed distinct differences between AL and PL where predominant MAG also changed with depth (Fig. 2, Fig. S1, Supporting Information). In the AL, the most abundant phyla were *Acidobacteria* and *Proteobacteria* while PL MAGs were dominated by *Actinobacteria, Bacteroidetes, Chloroflexi* and *Proteobacteria*. The most dominant MAGs in AL – maxbin2.039_sub (*Acidobacteria*), metabat.158 (*Proteobacteria*), metabat.89 (*Verrucomicrobia*) – declined to nearly undetectable levels in the PL. Members of *Proteobacteria*, *Verrucomicrobia* and *Chloroflexi*, were ubiquitous in PL and had similar abundances in the upper PL (PL1 and PL2) than deep PL samples (PL3 and PL4). We observed a decline in *Acidobacteria* and some *Actinobacteria* MAG abundances with depth. Previous 16S rRNA based analysis detected a single Actinobacteria family –*Intrasporangiaceae* – to be strongly dominant throughout the PL (Müller *et al*. 2018). However, we could not detect similar populations in this data set. We further examined both assembled contigs and un-assembled raw reads by Kaiju annotation and BBMAP alignment and found that *Intrasporangiaceae* constituted a relatively small portion of the contigs in assembled reads (1.2%) and in general of metagenomes as represented by raw reads (total of 3.3% in all metagenomes). More unique but highly represented MAGs were found in the deepest samples, like metabat.179 (*Chloroflexi*) in PL3 and metabat.151 (*Bacteroidetes*) in PL4. Likewise *Saccharibacteria*, candidate phylum *Dormibacteraeota (AD3)* and candidate phylum *Levybacteria* had their highest abundance in deep permafrost.

### Determining the complexities of the Svalbard permafrost by coverage-based groups

**Figure 3.**
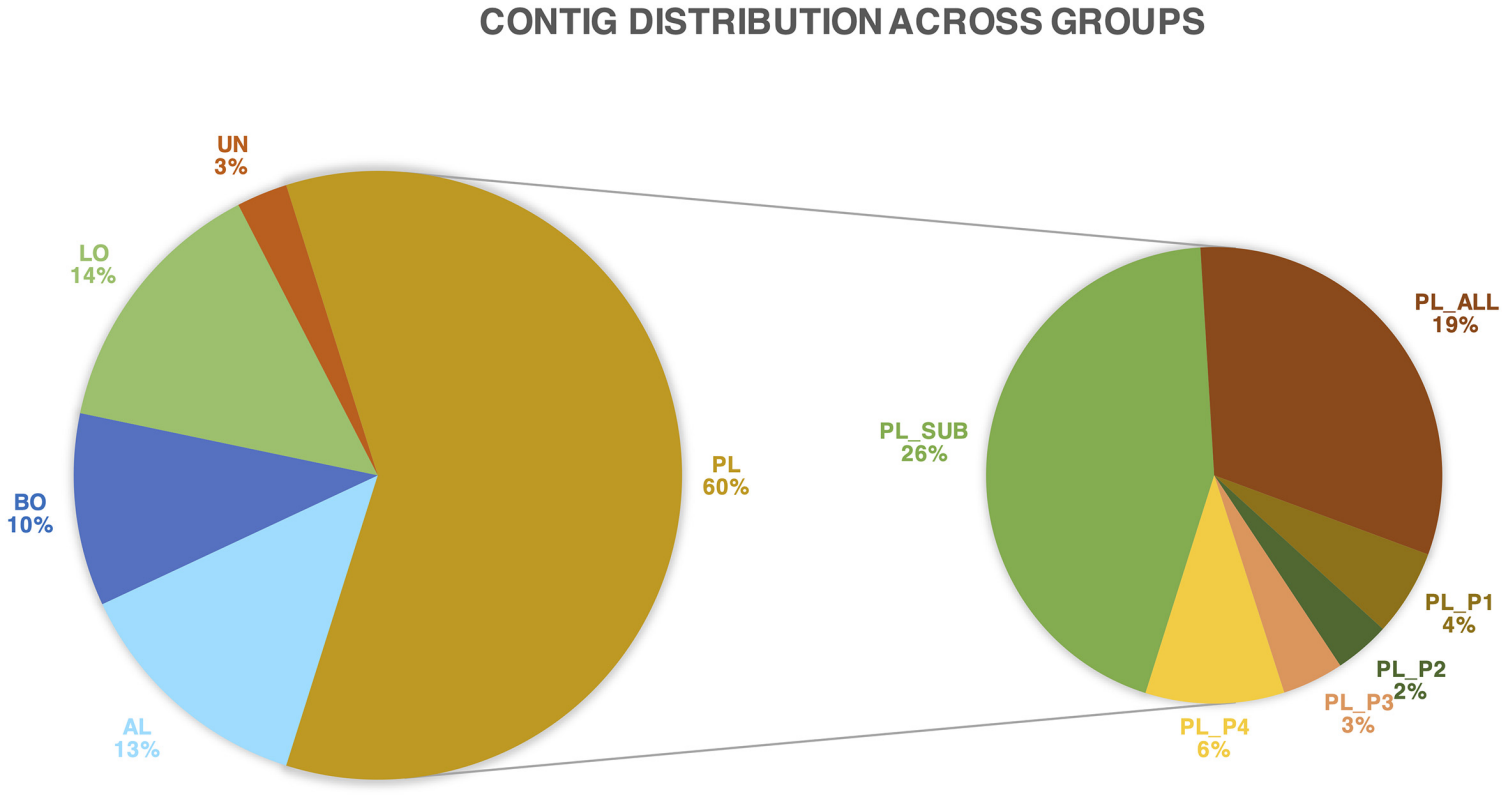
Contig distribution across groups. In total, 20573 contigs from all MAGs were assigned to each group based on pre-defined criteria (Table 1). KI (1%) and KD (5%) were not presented in the pie chart.

Many permafrost studies are focused on sample specific comparative analysis (Yergeau *et al*. 2010; Mackelprang *et al*. 2017; Müller *et al*. 2018), however, sample-based analysis is not able to reflect the complexity of microbial spatial arrangement directly. Moreover, we observed that there were some regular patterns in coverage distribution across multiple samples. To utilize to the maximum the information and enable a deeper understanding of permafrost microbial universe at a high-resolution, we developed a comparative strategy to investigate the variance of functional potential combing the genomic (coverage) and functional (KEGG) information in a MAG-centric view. Only contigs from MAGs were included in this analysis. 20,573 contigs originating from refined MAGs were assigned to classification groups (Table 1). PL group represented the largest portion of the data by covering 60% of the contigs (Fig. 3). About 10% of the contigs were shared between both AL and PL and ubiquitous at all samples (BO) while 13% of the contigs were only found in AL. After filtering 14% of low abundance contigs (LO), only 3% could not be assigned to any of the above groups (UN). Within PL 26% of the contigs fell under subset of PL (PL_SUB) category, 19% of the contigs was found in all 4 PL (PL_ALL) and represent the key functions in Svalbard permafrost. Only a small portion of the contigs were specific to each depth (a quarter of contigs were exclusively observed in only one sample (PL_P1, PL_P2, PL_P3, PL_P4) covering 2%–6% of the total contigs. We identified only a small fraction of contigs in PL_ALL and PL_SUB that had a strong correlation with depth profile: about 5% of contigs decreased (KD) and 1% function represented in contigs increased (KI) with depth. Group-based abundance distribution showed a clear distinct difference of dominant phylum among groups (Fig. S1, Supporting Information): *Acidobacteria* and *Proteobacteria* in AL; *Proteobacteria* in BO; *Actinobacteria*, *Bacteroidetes*, *Chloroflexi* and *Proteobacteria* in PL.

### Key metabolic functions governing carbon and nutrient cycles in Svalbard permafrost

**Figure 4.**
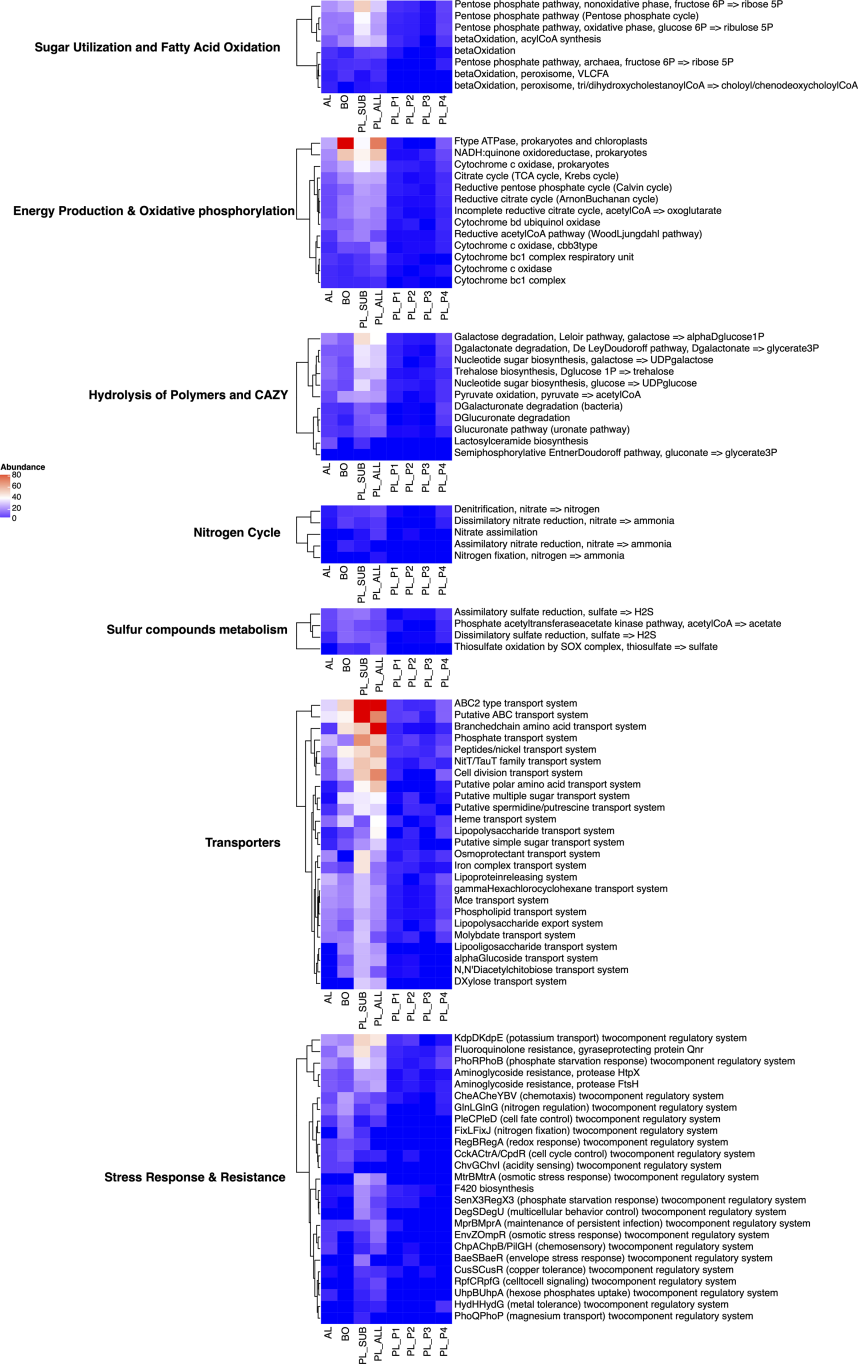
Trends in KEGG MO abundance in each group. The abundance of MO was calculated with normalized coverage in a MAG-centric strategy (see Methods).

About 451 out of 808 MO in the database were detected in Svalbard MAGs, several pivotal MO were selected and assigned into corresponding metabolic pathways manually, finally 8 pathways with 102 MO were retained (Table S3). Here we report MO of different pathways showed distinct abundance among groups (Fig. 4, Fig. S2).

#### Carbon cycling and energy production

We examined the trends in carbon cycle and energy production genes among different groups by focusing on hydrolysis of polymers, carbohydrate active enzymes (CAZY), sugar utilization, fatty acid oxidation, oxidative phosphorylation and energy production categories. One of the most abundant MO was F-type ATPase (F-ATPase), which was present in both BO and PL_ALL. This process is important because in Bacteria most ATP is produced by F-ATPase in the cytoplasmic membrane under aerobic conditions (otherwise by glycolysis and fermentation under anaerobic conditions) (Kühlbrandt 2019). MAGs belonging to group BO and PL_ALL also included a large number of aerobic respiratory chain complex modules, such as NADH: quinone oxidoreductase (NQR). Most living systems prefer to use conserved energy currencies, including proton motive force (PMF), NADH and ATP. NQR connects these energy currencies by using NADH produced during nutrient breakdown to generate a PMF, which is subsequently used for ATP synthesis (Barquera 2014). Collectively these trends show strong representation of aerobic respiratory processes in Svalbard permafrost, however, we also observed a decreasing trend in their abundance with depth (KD>KI, Fig. S2, Supporting Information). We further investigated dehydrogenases involved in fermentation, however, these were neither in high abundance nor showed strong grouping trends hence confirming the aerobic respiration as the dominant carbon cycling pathway in this location (Fig. S3, Supporting Information).

Polymer hydrolysis and CAZY functions were also found in abundance especially in core in PL groups (PL_ALL and PL_SUB). We found that galactose could be utilized to glucose (via Leloir) or to pyruvate (via De Ley) as both pathways were well represented in permafrost MAGs. Though a known bottleneck in Leloir is galactose transportation from outside of the cell, we also observed an over-representation of ABC transporters in PL group (Pathway: Transporters), which demonstrated the genetic potential of permafrost microbiomes to degrade galactose in carbohydrate metabolism. MAGs also showed potential to degrade more complex carbon sources all the way to CO_2_ (Figs S4 and S5, Supporting Information). For example, the most abundant MAG in this set *Chloroflexi* MAG metabat.179 (Genus: UBA5189) had xylulose kinase and xylose transporters (Table S4, Supporting Information), but lacked genes encoding xylose isomerase, the first enzyme of the isomerase pathway of xylose metabolism. Therefore, it was likely that only xylulose could be utilized. MAG metabat.179 also had three copies of GH3 family beta-hexosaminidase (chitinolytic) and related N-acetyl-D-glucosamine (GlcNAc) transporters. These enzymes can cleave monomers of GlcNAc from the non-reducing end of chitin oligomers. Additionally, this MAG contained a CO dehydrogenase and could use organic acids (L-Lactate dehydrogenase and Aconitate hydratase) hence showing the potential to utilize a range of polymeric carbon to CO_2_. Trehalose biosynthesis, a known carbon source and cryoprotectant, was also highly represented in PL (PL_ALL and PL_SUB). Pyruvate oxidation genes were found in both BO and PL indicating its importance for both AL and PL. We observed a decreasing trend (KD, Fig. S2, Supporting Information) in almost all polymer hydrolysis and CAZY functions except trehalose biosynthesis and pyruvate oxidation.

#### Nitrogen, methane and sulfur metabolisms

Within Svalbard MAGs nitrogen cycle was restricted to denitrification and dissimilatory nitrate reduction to ammonia. Both pathways were abundant in both BO and PL yet in comparison with other MOs, nitrogen cycling genes constituted a small portion of the genetic potential. Even so, some MAGs, like Bacteroidetes MAG metabat.151, showed a potential of full denitrification (Fig. S6, Supporting Information). We did not detect MO and genes involved in nitrification. At least one copy of glutamine synthetase (EC 6.3.1.2), glutamate synthase (EC 1.4.1.13) and ammonium transporters (Amt) were found in most abundant MAGs and were also well represented in both AL, BO and PL groups. All together, these results show the potential to use organic nitrogen and available ammonia in the environment through the depth profile in Svalbard soils. In this set only *Firmicutes* MAG maxbin2.064_sub (Genus: *Desulfosporosinus*) was found to be capable of nitrogen fixation, whereas another key biogeochemical process methane metabolism was not found in Svalbard MAGs.

Genes for dissimilatory sulfite reduction, the sulfur oxidation (SOX) gene complexes mediating thiosulfate oxidation and assimilatory sulfite reductase MOs were present in Svalbard MAGs. These MOs were in low abundance, but internal comparison among the groups revealed distinct trends. For example, assimilatory sulfate reduction was abundant in all groups while dissimilatory sulfate reduction had its strongest trend in PL. However, we also detected co-occurrence of these pathways. For example, one of the most abundant MAGs, *Proteobacteria* MAG maxbin2.012 (Genus: *Gallionella*) contained genes involved both in assimilatory and dissimilatory sulfate reduction (Figs S7 and S8, Supporting Information). Additionally, thiosulfate oxidation by SOX complex was found mainly dominant in PL_ALL. This complex has been shown to produce either sulfate (complete pathway) or elemental sulfur (incomplete pathway) in diverse organisms (Houghton *et al*. 2016). We detected a decreasing trend (KD, Fig. S2, Supporting Information) in assimilatory sulfate reduction with increasing depth but not with dissimilatory sulfite reduction. These findings underlined the importance of ability to metabolize sulfur in Svalbard MAG lifecycle.

#### Stress responses and antibiotic resistance

Permafrost microorganisms have reportedly been shown to contain a suite of systems to deal with environmental stressors, such as cold-shock proteins and osmotic stress proteins, to counter the extreme physical and chemical stresses, including freezing temperatures, oligotrophic conditions and high salinity (Mackelprang *et al*. 2016a). We observed enrichment of KdpDE: potassium transport system in PL (PL_ALL and PL_SUB), which is required for maintaining the intracellular pH by buffering the negative charge of amino acids and used in many bacteria as a compatible solute to counteract osmotic stress (Gundlach, Commichau and Stülke 2018). Additionally, we found several two component regulatory transport systems involved in cell processes and cycle control, redox response and chemotaxis in high abundance in PL (PL_ALL and PL_SUB). Another major stress response MO was phosphate starvation response system (PhoR−PhoB), which was highly abundant in PL (PL_ALL and PL_SUB) groups, especially in PL4. Concomitantly, phosphate transport systems were among highly abundant transporters shared between AL and PL groups. These findings indicate that regulation intracellular pH and phosphorus availability are pivotal for Svalbard MAGs.

Besides MO managing environmental stressors, several antibiotic resistance genes acting against aminoglycosides and fluoroquinolones were highly abundant in PL. The aminoglycosides are natural antibiotics produced by soil bacteria where broad-spectrum bactericidal activity is achieved by interference with protein synthesis, including corruption of the genetic code via bind to rRNA and proteins within the 30S subunit of the ribosome (Cox *et al*. 2015). Fluoroquinolones are another class of broad-spectrum antibiotics that target the type II topoisomerases (DNA gyrase and topoisomerase IV) involved in the maintenance of DNA topology (Rutgersson *et al*. 2014). In a previous work, Qnr has been found as a novel mechanism of natural fluoroquinolones resistance in bacteria (Chen *et al*. 2013).

## DISCUSSION

Complexity and unmatched diversity in soil metagenomes provide many challenges to data analysis; especially to those seeking to recover high-quality MAGs. DASTool (Sieber *et al*. 2018), a recently published bin refinement tool, aims to recover more near-complete genomes by aggregating and integrating bins generated from established binning algorithms (Kang *et al*. 2015; Wu, Simmons and Singer 2016). Applications of DASTool (Danczak *et al*. 2019; Imperato *et al*. 2019; Seitz *et al*. 2019) showed significantly improved MAG refinement and recovery. Yet when reconstructing permafrost MAGs these efforts might still not be sufficient. For example, in this study we observed that 21 out of 64 metagenome bins remained highly contaminated (> = 10%) even after using DASTool. We developed a script to recover bins that would be otherwise discarded (Fig. 1A). While several bin refinement strategies are deployed by IMG/M (Chen *et al*. 2019) and Anvi'o (Eren *et al*. 2015) our workflow provides a scalable and flexible alternative where thousands of bins could be analyzed systematically. We picked Kaiju as taxonomic classifier due to its extensibility as it provides fast and sensitive annotations of large contig sets. With our script, the user can choose different taxonomic reference databases – such as RefSeq, NCBI nr database or local – depending on their research goals. More importantly, contaminated contigs could be detected at all taxonomic levels and bins could be refined up to species level. Our script traces the hierarchical relationships using a user defined percentage threshold and subset contaminated bins for all ranks from phylum to species level. Removing possible contaminated contigs from a MAG may reduce completeness in some cases due to the inaccuracy in the taxonomic assignments. With our improved workflow for MAG refinement, we successfully reported 56 out of 64 MAGs with low contamination (< = 10%).

Here, we also developed a new comparative strategy for investigating functional potential based on coverage with a MAG-centric view (Fig. 1B). Generally, metagenomic functional analysis was achieved by mapping short reads or assembled contigs with predicted genes against reference databases followed by parsing the result in gene or pathway level approaches (Mackelprang *et al*. 2017; Müller *et al*. 2018). Gene-by-gene approaches utilizes most dominant gene products while overlooking the fact that biological functions rely on multiple genes while only a subset of them may be significantly abundant. For another, pathway-level analysis can miss nuanced differences in functional variance as a key pathway could contain many shared sub-pathways or genes. Motivated by this, we deployed a comparative analysis strategy that utilizes KEGG Module, a collection of manually defined functional units each encompassing a set of genes – represented by KO identifiers (Kanehisa *et al*. 2012). Comparing with pathway or gene enriched analysis, module-based analysis directly links to specific metabolic capacity (Kanehisa *et al*. 2014). Coverage is another important metagenomic characteristic (Albertsen *et al*. 2013; Sharon *et al*. 2013; Quince *et al*. 2017) that is currently not used beyond binning assembled contigs into MAGs (Alneberg *et al*. 2014; Imelfort *et al*. 2014; Kang *et al*. 2015; Wu, Simmons and Singer 2016). Our approach takes into account coverage and patterns of presence/absence and changes in coverage between samples through defining profiles or groups (Table 1) and analyzing KEGG Module-based functional information across these groups. In Svalbard permafrost this approached allowed identification of functions linked with depth in addition to aiding capture of new trends distinguishing AL and PL (Fig. 4). Although we have focused on permafrost metagenomics in this work, strategies similar to those applied here are applicable to other metagenomic studies, especially for well-characterized environments such as human gut with more accurate taxonomic classification and available MAGs as well as additional information on samples.

Svalbard soil and PLs were previously described via 16S rRNA gene amplicon sequencing up to a depth of 2 m where microbial communities in PL were dominated by the *Actinobacteria* (family *Intrasporangiaceae*). *Intrasporangiaceae* 16S rRNA gene was found in an average abundance of 70% in PL; however, we only found this group to account for 3.3% of the all raw reads and 1.2% of assembled contigs. This could be caused by differences in biases between the two sequencing methodologies. Currently sequenced *Intrasporangiaceae* genomes (JGI IMG/M) contain 1–5 copies of 16S rRNA gene which could cause an overestimation when analyzed via amplicon sequencing. Another reason for this mismatch can originate from under-sampling of *Intrasporangiaceae* populations during metagenome sequencing. *Intrasporangiaceae* genomes are really high-GC content populations (68%–74% of GC range 63 genomes in JGI IMG/M), hence such high-GC rich fragments can be under-sampled during metagenomic library preparation, fail to pass quality checks during base calling and have difficulties during assembly (Bowers *et al*. 2015).

The grouping approach proposed here enabled us to determine key functions and trends in different cell and biochemical cycles propagated by each MAG through a permafrost depth profile. The most strikingly abundant microbial metabolism in this set of MAGs was aerobic. Vertical soil profiles are often depicted as aerobic zones transitioning neatly into anaerobic zones where terminal electron accepting processes and fermentation govern carbon decomposition (Mackelprang *et al*. 2016b). Yet soil systems, especially permafrost, are shown to be more complex. In permafrost aerobic microsites can exist within ice where low-to-freezing temperatures enable oxygen transfer into water (Jansson and Taş 2014). Via use of ^14^C-acetate and ^14^C-glucose microbial communities in permafrost from Canadian high Arctic were shown to be active at near ambient subzero temperatures (−5°C to −15°C) (Steven *et al*. 2008). More recently activity of both tundra and permafrost microbes at subzero temperatures were shown via stable isotope probing (Tuorto *et al*. 2014; Gadkari *et al*. 2019). Carbon degradation pathways identified in cold soils and permafrost show abundance and activity of various aerobic and anaerobic pathways at different locations. Genes involved in starch, lignocellulose, chitin, cellulose and trehalose degradation in both the active layer and permafrost (Yergeau *et al*. 2010; Mackelprang *et al*. 2011; Gadkari *et al*. 2019) and anaerobic metabolism was identified as a common microbial trait in permafrost metagenomes (Lipson *et al*. 2013; Hultman *et al*. 2015; Woodcroft *et al*. 2018). Our current knowledge of intact and thawing permafrost points to a large variance in metabolic potential and its utilization among different geographical locations (Mackelprang *et al*. 2016b). In Svalbard permafrost, we found aerobic processes as the key metabolism (Fig. 4) of recovered MAGs which showed previously unreported metabolic potential in permafrost. Besides genes involved SOM degradation (Fig. S3, Supporting Information), we found that in permafrost MAGs for aerobic processes dominating cellular metabolism. These results indicate that a substantial investment by permafrost MAGs in energy production is required to maintain reactions in order to survive at low temperatures. These results are also in concurrence with previous activity measurements from the same location where through a series of incubations Müller *et al*. (Müller *et al*. 2018) showed upon permafrost thaw up to four times higher CO_2_ respiration rate were observed under aerobic than anaerobic conditions. Additionally, permafrost samples emitted similar quantities of CO_2_ to active layer soils suggesting that Svalbard permafrost microbiome can stimulate its aerobic metabolism upon thaw. CH_4_ is an important component of soil GHG fluxes in the Arctic which is shown to be released upon permafrost thaw as a result of significant changes in microbial populations and their interactions (Singleton *et al*. 2018; Woodcroft *et al*. 2018). In this study; however, we did not find any methanogenic MAGs or methane oxidation potential genes and anaerobic incubation experiments yielded no CH_4_ production (Müller *et al*. 2018).

Arctic soils and permafrost are nitrogen limited where importance of nitrogen fixation for permafrost microbiome was highlighted by earlier metagenomics efforts (Yergeau *et al*. 2010; Mackelprang *et al*. 2011). It was hypothesized that the frozen conditions in permafrost sequester biologically available nitrogen, making nitrogen fixation necessary to contain metabolic activity. Hultman *et al*. (Hultman *et al*. 2015) showed that the permafrost microbiome was poised to assimilate nitrogen where genes encoding both *glutamine-* and *glutamate synthases* were transcribed and translated in permafrost. These pan-arctic observations were also paralleled in Svalbard active layer soils where Schostag et al (Schostag *et al*. 2015) detected high abundance of nitrogen-fixing bacteria via 16S rRNA gene sequencing. Svalbard permafrost MAGs showed similar trends to these previous findings where throughout the depth profile most abundant MAGs had *glutamine synthetase*, *glutamate synthase* and ammonium transporters to assimilate nitrogen. Earlier research showed that 450–550 μg/L ammonia could be found in Svalbard permafrost layers (Müller *et al*. 2018). In contrast, nitrogen fixation potential was limited, which collectively suggest nitrogen limitation as an important constraint to cellular activity in intact and thawed permafrost.

Sulfur metabolism have been shown to be widely present in permafrost microbes (Hansen *et al*. 2007; Vatsurina *et al*. 2008; Lipson *et al*. 2013; Chauhan *et al*. 2014). While sulfite reduction and sulfur oxidation were found in permafrost at different depths (Jansson and Taş 2014; Hultman *et al*. 2015), sulfate reduction rates were only high in bog samples while almost negligible in intact permafrost (Hultman *et al*. 2015). Current knowledge from metagenome data suggest that redox conditions become favorable for sulfate reduction after permafrost thaw. Svalbard MAGs provide a new perspective to sulfur metabolism in permafrost where abundant MAGs to contained genes involved both in assimilatory and dissimilatory sulfate reduction (Figs S7 and S8, Supporting Information). Genomic evidence suggests that *Gallionella* (one of the main sulfur cycle MAGs: maxbin2.012) are adapted to extremely low oxygen levels, it is possible that they are capable of growth at dissolved O_2_ concentrations below the oxygen detection limits to occupy a narrow niche between O_2_ and redox gradients (Emerson *et al*. 2013; Berg *et al*. 2019). We hypothesize that Svalbard MAGs retain flexibility in their sulfur metabolism in order to fully utilize limited resources propagated by ice and formation or microsites.

Genes involved in stress responses, resistance and resilience are shown to be crucial part of not only permafrost microorganisms but also psychrophiles in general (Ayala-Del-Río *et al*. 2010; Mykytczuk *et al*. 2013). Microbial survival in permafrost is challenging: proteins are less flexible and are prone to denaturation (Mykytczuk *et al*. 2013), cell membranes often susceptible to lose their fluidity (Ayala-Del-Río *et al*. 2010), water retention can be challenging and nutrient transport can be constrained. As a result, efficient anion and cation transporters is beneficial for cell survival. We observed an enrichment of potassium transport regulatory system in abundant permafrost MAGs (Fig. 4). The presence of potassium transporter protein in permafrost was also confirmed by a previous metaproteomics study (Hultman *et al*. 2015). As these transporters serve an important role in maintaining the intracellular pH, counteract osmotic stress and also required as cofactors for many enzymes. Finally, potassium is essential for the activity of many enzymes and protein complexes including the ribosome as well as for the regulation of gene expression. Their enrichment in MAGs shows high capability in regulating cellular functions and potential activity in frozen soils. Hultman *et al*. (Hultman *et al*. 2015) found high numbers of cold-shock proteins in permafrost. Though present in Svalbard MAGs cold-shock proteins were not highly abundant in MAGs; instead cell fate and cycle control, redox response and chemotaxis regulatory systems were of high abundance. Transmembrane receptors are ubiquitously used by prokaryotes in environmental sensing (Bi, Jin and Sourjik 2018). As a result, it can be expected that cellular functions controlling these systems are retained and maybe enriched in permafrost. Surprisingly we did not identify spore forming potential as a key functional potential of Svalbard MAGs. This in line with the previous assessment that spores are not the best survival strategy for freezing conditions (Mondav *et al*. 2014). Besides environmental stressors, several antibiotic resistance genes acting against aminoglycosides and fluoroquinolones were among key functions shared among Svalbard permafrost MAGs. Antibiotic resistant bacteria were found both among the Arctic and Antarctic isolates (Mindlin and Petrova 2017) where about one third of the isolated permafrost strains were resistant to more than one antibiotic. Aminoglycosides were observed in ancient permafrost samples as well (Dcosta *et al*. 2011; Kashuba *et al*. 2017). Resistance against fluoroquinolones, which directly inhibit DNA synthesis, is a widespread microbial survival strategy (Rutgersson *et al*. 2014). Antibiotic resistance is an inherent property of permafrost microbiome however we are yet to understand the importance of these mechanisms on permafrost microbial diversity and biochemical cycles beyond their apparent role in survival.

Svalbard MAGs carry signatures of metabolic pathways that provide tight control of growth and resources. Almost all living cells sophisticatedly regulate their phosphate uptake that enables survival under phosphate-limiting conditions (Marzan and Shimizu 2011). In particular, regulation of phosphate may play an important role when nitrogen is also limiting. We found that metabolism involved in recycling and acquisition of ammonium was concomitant with strong representation phosphate regulation (i.e. starvation response and related transporters). Especially in phosphate depleted soils efficient phosphorus transporters are pivotal, as they allow microorganisms to compete for bioavailable phosphorus. Here, we hypothesize that microbial growth, survival and diverse metabolism including energy and central carbon cycling in Svalbard permafrost is facilitated by coupled regulation of ammonium, sulfur and phosphate metabolism. Even though we are not able to tie this hypothesis to availability of nutrients or gene expression that regulates these metabolisms, it is tempting to speculate that under freezing conditions Svalbard microbial populations regulate extra- and intra-cellular nutrient stoichiometry and availability closely to survive and utilize a wide range of carbon resources.

## CONCLUSIONS

Predicting metabolic functionality and responses to changing environmental conditions from metagenomic data are among the greatest challenges in microbial ecology today (Myrold, Zeglin and Jansson 2014). Still metagenomics can be used to generate novel hypotheses about microbial metabolism and lifestyle. Permafrost in Svalbard is predicted to be more sensitive to increases in soil temperature and active layer thickness than the permafrost of extensive lowlands in Siberia, northern Canada and Alaska. In addition, Svalbard is an archipelago located near the northern most branches of the North Atlantic Current and the southern limit of the polar icepack. Even small variations in these important phenomena will induce rapid climatic variations with potential effects on the local Svalbard climate and permafrost. In this study, we provide an in-depth analysis of key permafrost microbial functions in Svalbard via a MAG-centric analysis. Svalbard MAGs were mostly aerobic and showed enrichment in functions regulating ammonium, sulfur and phosphate metabolism. Among different permafrost depths we repeatedly observed these metabolic pathways. Their perseverance point to their potential importance to life in permafrost. Our analysis also identified effective resource acquisition from the environment in potentially competitive and limited resource conditions as a key permafrost microbiome property. Collectively our results showed that Svalbard MAGs contain previously unreported metabolic functions in a permafrost environment.

## DATA AND CODE AVAILABILITY

The shotgun sequence data and recovered MAGs were deposited in the European Nucleotide Archive (ENA) database under the study number PRJEB30872.

An instruction of refining MAGs and source code is available at https://github.com/yxxue/Recovery-and-refinement-of-MAGs-for-permafrost-metagenome.

A demonstration of comparative functional analysis by coverage in Svalbard metagenome and related source code are available at https://github.com/yxxue/Coverage-based-functional-analyisis-in-a-MAG-centric-view.

## Supplementary Material

fiaa057_Supplemental_Files
