# Supplementary material for: Metagenome-assembled genome distribution and key functionality highlight importance of aerobic metabolism in Svalbard permafrost
Source: FEMS Microbiol Ecol. 2020 Apr 17;96(5):fiaa057. doi: 10.1093/femsec/fiaa057 (PMC7174036; doi:10.1093/femsec/fiaa057)
Supplement: fiaa057_Supplemental_Files [file fiaa057_supplemental_files.zip › Supplementary_data_info.docx]

**Figure S1: The relative abundance of MAGs represented among main groups.** Three dominant sample groups are presented: AL, BO, and PL.

**Figure S2: Heatmap shows the abundance of selected KEGG MO between KI (Increasing trend in PL_ALL and PL_SUB) and KD (Decreasing trend in PL_ALL and PL_SUB).** KI and KD represent contigs with strong correlations (KI:>= 0.9, KD<= -0.9) between depth (cm: 110, 122, 135, 170) and normalized coverage in PL samples.

**Figure S3: Alcohol Dehydrogenase at Svalbard MAGs among sample groups.** The bar chart shows abundance distribution of KEGG Orthology (KO) related with Alcohol Dehydrogenase metabolism among groups in a MAG-centric view.

**Figure S4: Cellulotic Enzymes at Svalbard MAGs among sample groups.** The bar chart shows abundance distribution of KEGG Orthology (KO) related with Cellulotic Enzymes metabolism among groups in a MAG-centric view.

**Figure S5: CO Dehydrogenase at Svalbard MAGs among sample groups.** The bar chart shows abundance distribution of KEGG Orthology (KO) related with CO Dehydrogenase metabolism among groups in a MAG-centric view.

**Figure S6: Nitrogen cycle at Svalbard MAGs among groups.** The bar chart shows abundance distribution of KEGG Orthology (KO) related with Nitrogen cycle metabolism among groups in a MAG-centric view.

**Figure S7: Dissimilatory sulfate reduction at Svalbard MAGs among groups.** The bar chart shows abundance distribution of KEGG Orthology (KO) related with Dissimilatory sulfate reduction metabolism among groups in a MAG-centric view.

**Figure S8: Assimilatory sulfate reduction at Svalbard MAGs among groups.** The bar chart shows abundance distribution of KEGG Orthology (KO) related with Assimilatory sulfate reduction metabolism among groups in a MAG-centric view.

**Table S1: Taxonomic classification of MAGs.**

**Table S2: 16S rRNA comparative analysis of Svalbard MAGs with recent stable isotope probing studies.**

**Table S3: Selected KEGG Modules (MO) and their corresponding pathways.**

**Table S4: Key SOM degradation genes in Chloroflexi MAG metabat.179.**
