# Supplementary figures and images for: Metagenome-assembled genome distribution and key functionality highlight importance of aerobic metabolism in Svalbard permafrost
Source: FEMS Microbiol Ecol. 2020 Apr 17;96(5):fiaa057. doi: 10.1093/femsec/fiaa057 (PMC7174036; doi:10.1093/femsec/fiaa057)

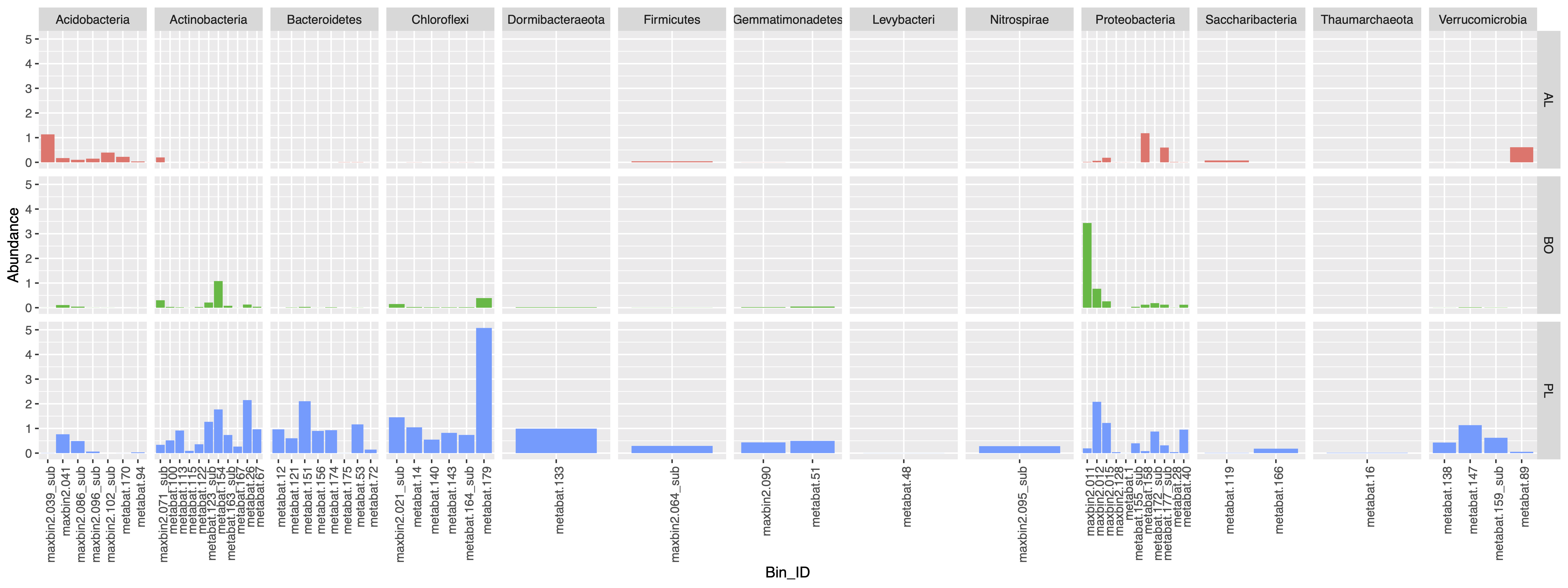

Supplement: fiaa057_Supplemental_Files [file fiaa057_supplemental_files.zip › Figure_S1.tiff]

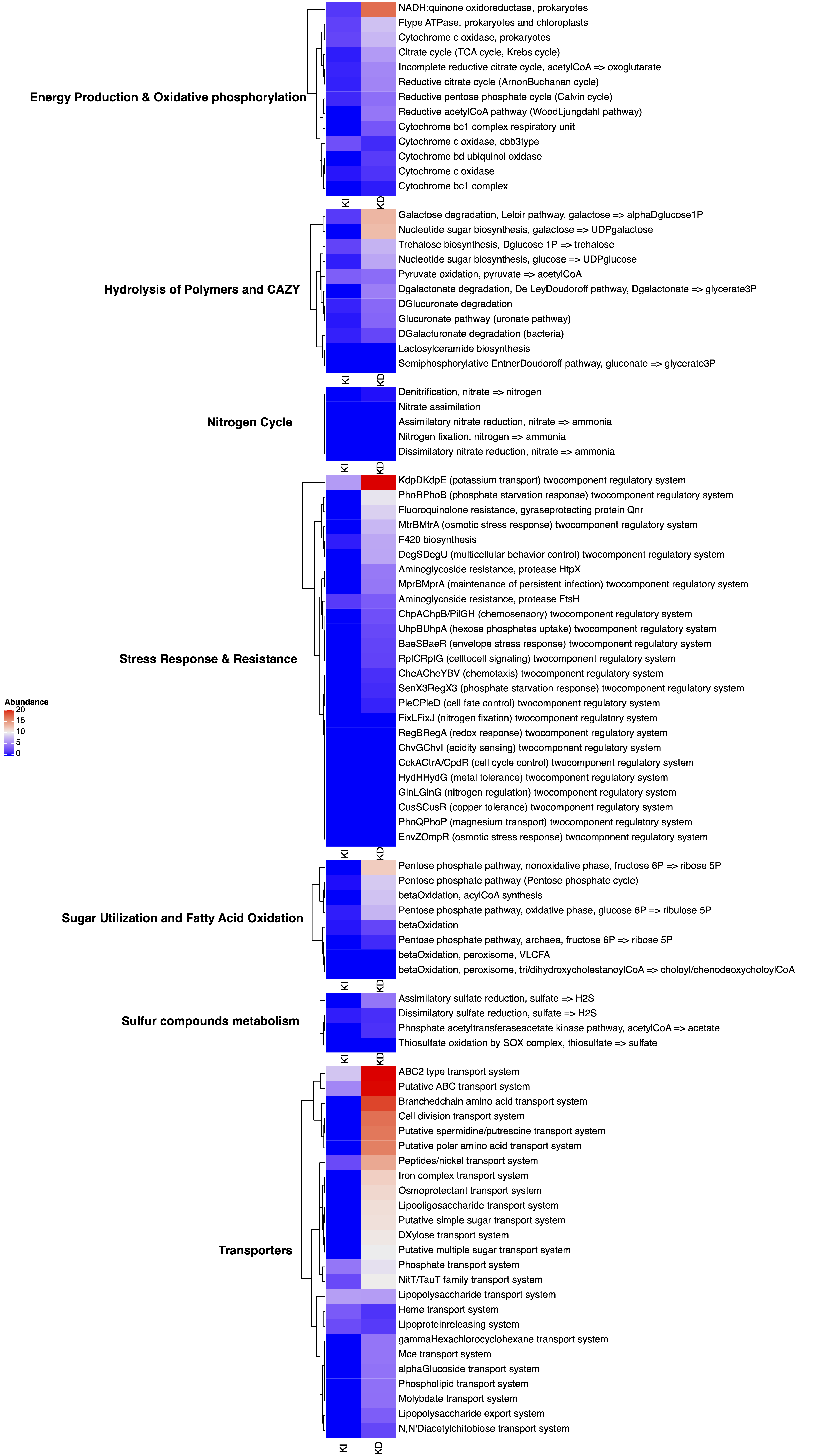

Supplement: fiaa057_Supplemental_Files [file fiaa057_supplemental_files.zip › Figure_S2.tiff]

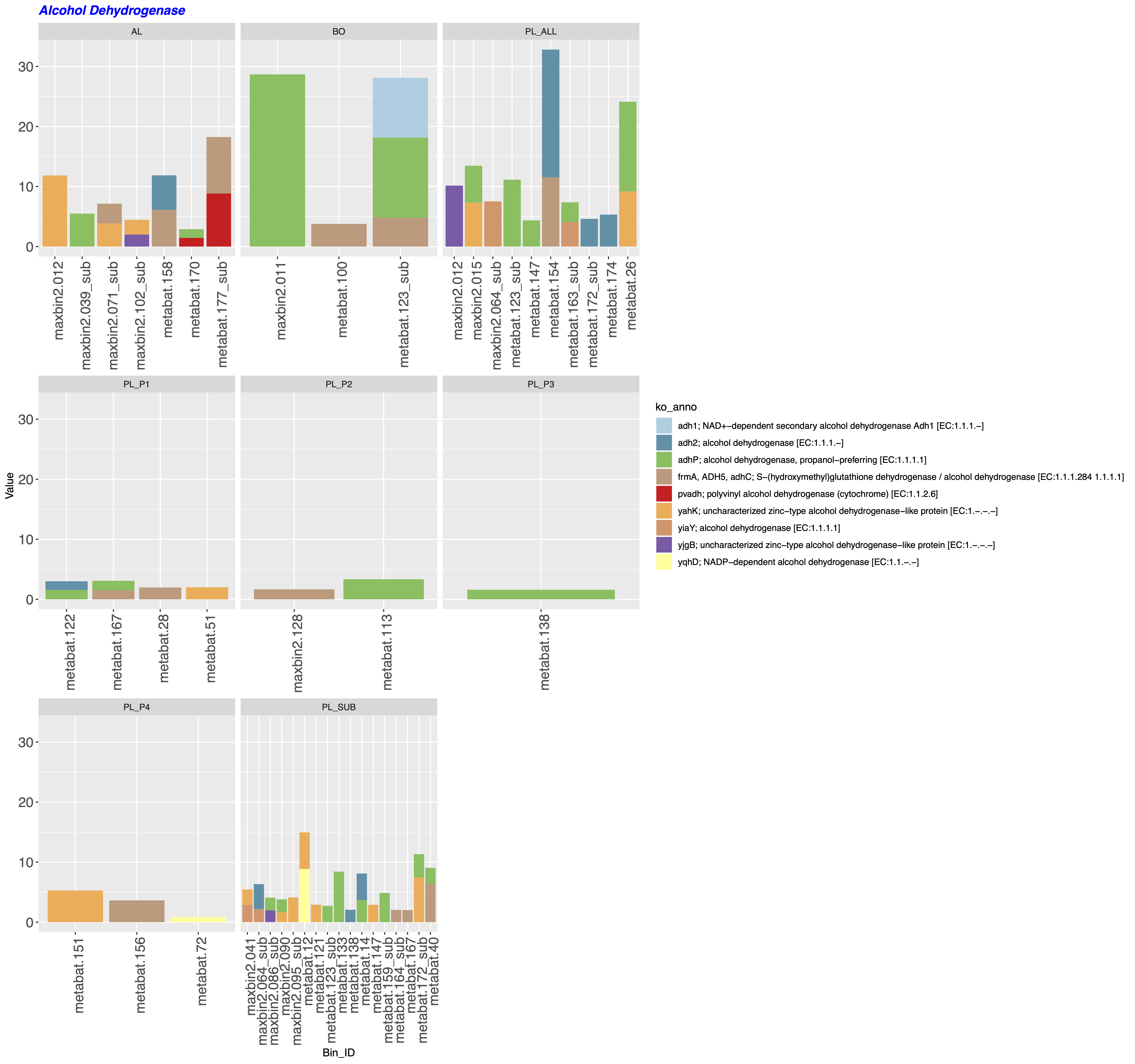

Supplement: fiaa057_Supplemental_Files [file fiaa057_supplemental_files.zip › Figure_S3.tiff]

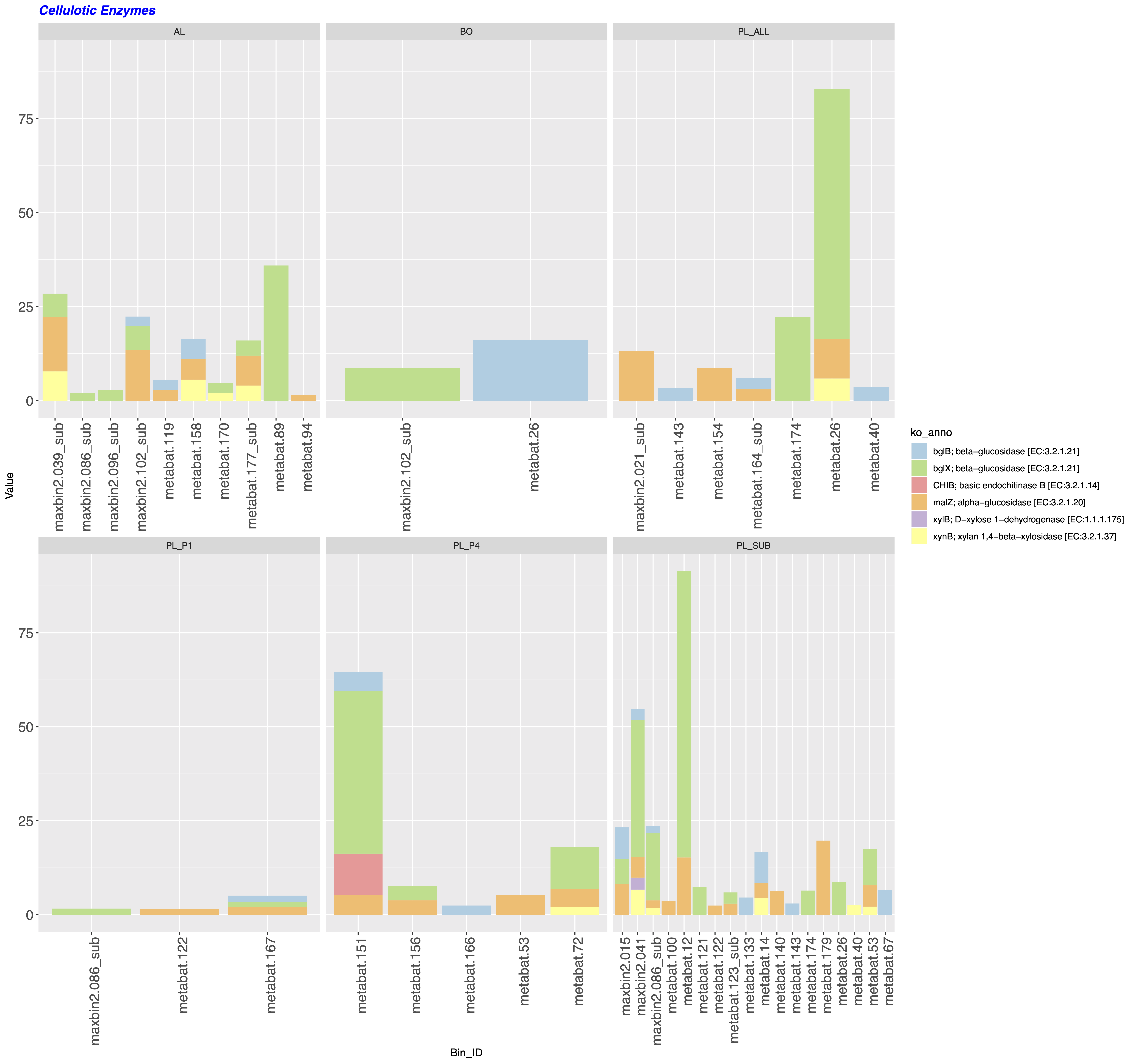

Supplement: fiaa057_Supplemental_Files [file fiaa057_supplemental_files.zip › Figure_S4.tiff]

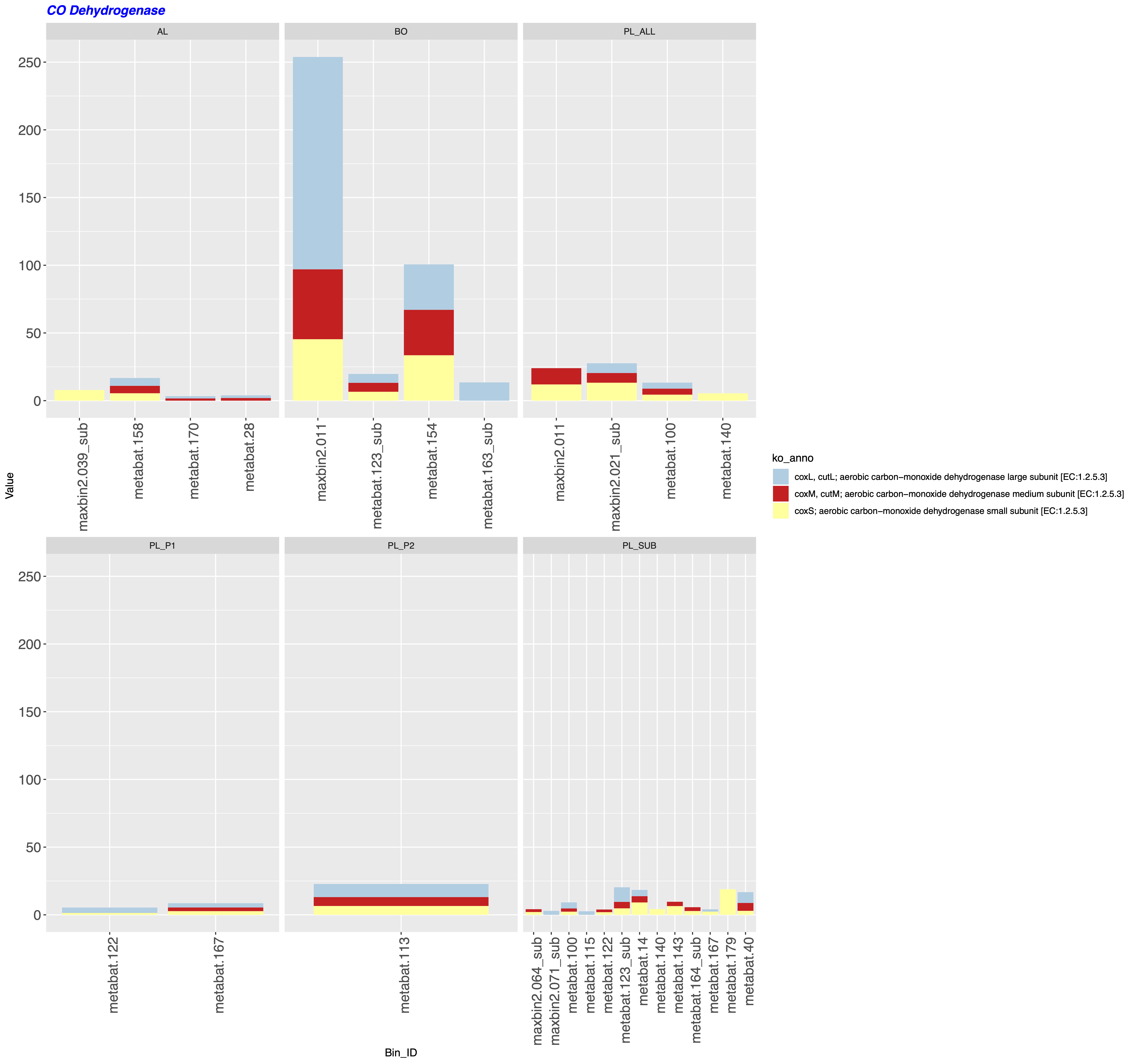

Supplement: fiaa057_Supplemental_Files [file fiaa057_supplemental_files.zip › Figure_S5.tiff]

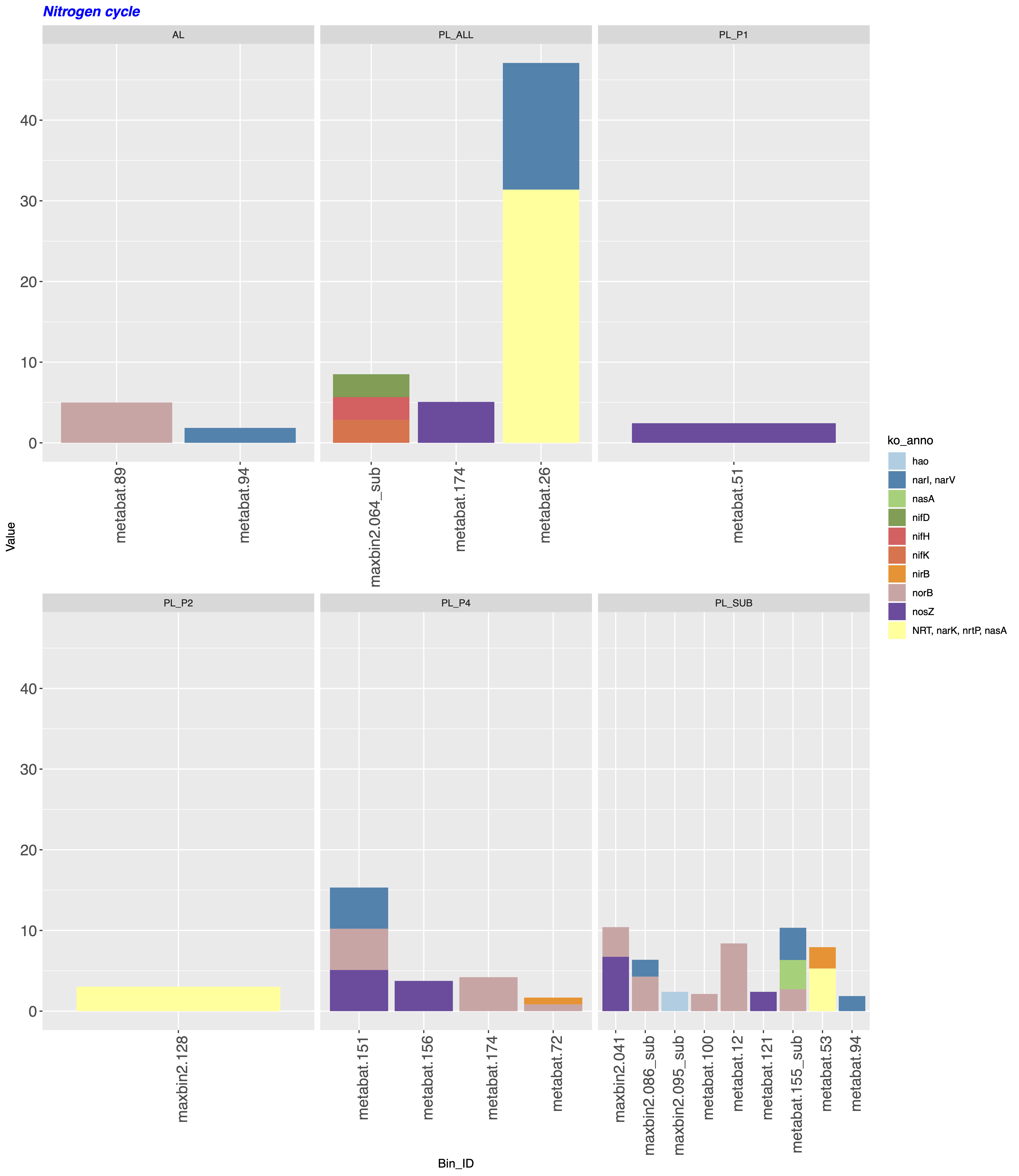

Supplement: fiaa057_Supplemental_Files [file fiaa057_supplemental_files.zip › Figure_S6.tiff]

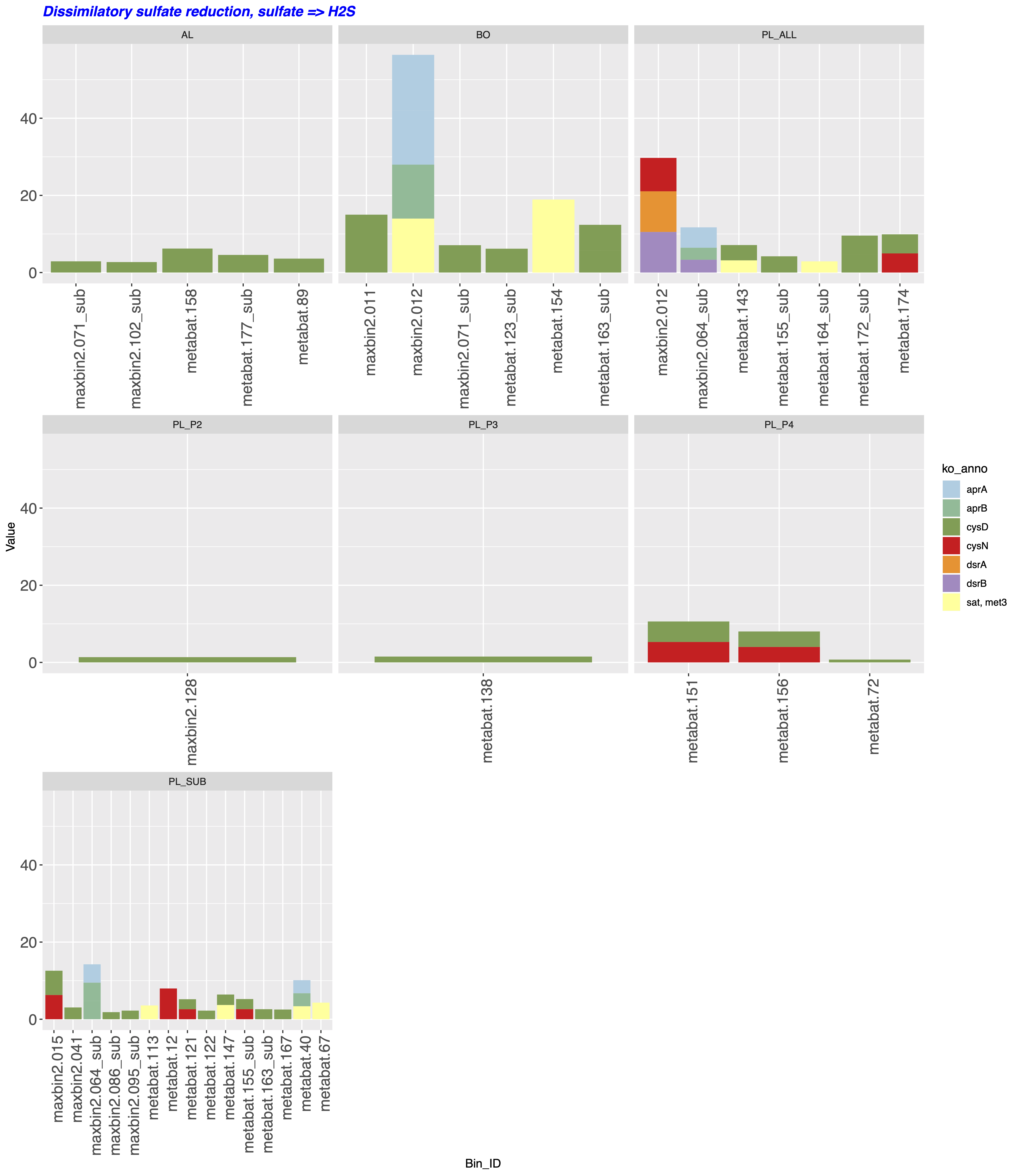

Supplement: fiaa057_Supplemental_Files [file fiaa057_supplemental_files.zip › Figure_S7.tiff]

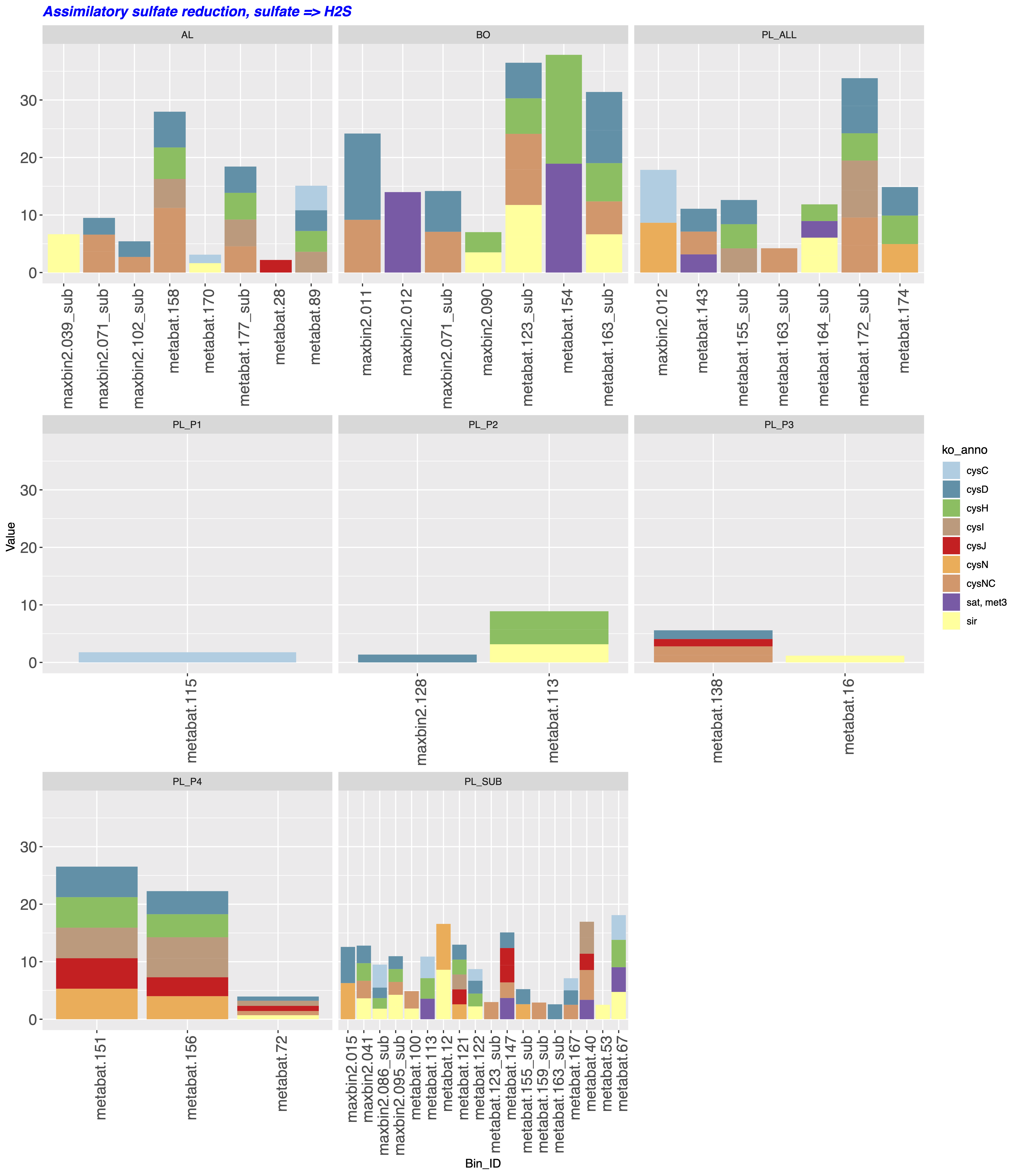

Supplement: fiaa057_Supplemental_Files [file fiaa057_supplemental_files.zip › Figure_S8.tiff]
